# A Flexible Multiring Concentric Electrode for Non-Invasive Identification of Intestinal Slow Waves

**DOI:** 10.3390/s18020396

**Published:** 2018-01-30

**Authors:** Victor Zena-Giménez, Javier Garcia-Casado, Yiyao Ye-Lin, Eduardo Garcia-Breijo, Gema Prats-Boluda

**Affiliations:** 1 Centro de Investigación e Innovación en Bioingeniería, Universitat Politècnica de València, Valencia 46022, Spain; vfzena@ci2b.upv.es (V.Z.-G.); yiye@ci2b.upv.es (Y.Y.-L.); gprats@ci2b.upv.es (G.P.-B.); 2 Instituto Interuniversitario de Investigación de Reconocimiento Molecular y Desarrollo Tecnológico, Universitat Politècnica de València, Valencia 46022, Spain; egarciab@eln.upv.es

**Keywords:** ring electrodes, surface recording, Laplacian recordings, intestinal slow wave

## Abstract

Developing new types of optimized electrodes for specific biomedical applications can substantially improve the quality of the sensed signals. Concentric ring electrodes have been shown to provide enhanced spatial resolution to that of conventional disc electrodes. A sensor with different electrode sizes and configurations (monopolar, bipolar, etc.) that provides simultaneous records would be very helpful for studying the best signal-sensing arrangement. A 5-pole electrode with an inner disc and four concentric rings of different sizes was developed and tested on surface intestinal myoelectrical recordings from healthy humans. For good adaptation to a curved body surface, the electrode was screen-printed onto a flexible polyester substrate. To facilitate clinical use, it is self-adhesive, incorporates a single connector and can perform dry or wet (with gel) recordings. The results show it to be a versatile electrode that can evaluate the optimal configuration for the identification of the intestinal slow wave and reject undesired interference. A bipolar concentric record with an outer ring diameter of 30 mm, a foam-free adhesive material, and electrolytic gel gave the best results.

## 1. Introduction

### 1.1. Biosignal Recordings on the Body Surface

The non-invasive recording of electrophysiological signals is commonly used in several fields of applied medicine, such as electrocardiography, electroencephalography and electromyography of many other organs and muscles. Although these recordings are usually made with conventional disc electrodes, these yield poor spatial resolution, caused in part by the blurring effect of the different conductivities of the volume conductor [1,2].

The Laplacian potential has been demonstrated to reduce this effect and provide better spatial resolution. Surface Laplacian potentials can be estimated by applying discretization techniques [3,4] and combining the surface potentials picked up by a set of monopolar electrodes, usually with a central electrode and four additional electrodes in the form of a square [3], or alternatively three electrodes in a triangle [4]. On the other hand, concentric ring electrodes of different sizes and configurations (bipolar, tripolar, tripolar connected in bipolar configuration) have been designed and tested to obtain a direct estimation of the Laplacian potential [5,6,7]. However, as these electrodes are mostly developed on rigid substrates, they cannot properly adapt to the body curvature and so can cause discomfort to the patient and also require to be fixed in place by external adhesive. The tripolar recording configuration is more spatially sensitive with a smaller amplitude than the bipolar. When signal amplitude is more important than spatial specificity, then the bipolar configuration appears superior [2,8]. Regarding electrode dimensions, it is known that the larger the electrode the wider the recording area, the deeper the sources that can be sensed and the higher the signal amplitude, but also the poorer the spatial selectivity [8]. The electrode dimensions should therefore be optimized for specific applications. In this regard, one of the objectives of this work was to develop a versatile electrode that admits multiple configurations with different ring sizes to study the best sensing setup for different bioelectrical recording applications. To facilitate its clinical use, the developed electrode is implemented onto a flexible substrate, is self-adhesive (two adhesive materials were tested with and without foam), incorporates a single connector and can perform dry or wet (with gel) recordings.

### 1.2. Intestinal Myoelectrical Activity

The electroenterogram (EEnG) is a record of the myoelectric activity of the small intestine. It has two components: the slow wave (SW) and the spike bursts (SB). The SW is associated with the slow and periodic oscillations of the basal signal. It is the intestinal pacemaker and determines the maximum frequency of intestinal contractions associated with the SB. The frequency of the SW varies along the small intestine, from around 12 cycles per minute (cpm) in the duodenum to 9 and 11 cpm in the jejunum and about 8 cpm in the ileum [9,10,11]. Although abnormal SW patterns are related with diabetes [12] and several intestinal pathologies, such as mechanical intestinal obstruction, irritable bowel syndrome, paralytic ileus, and intestinal ischemia [13,14], surface EEnG recording is not yet used in clinical practice. One of the main reasons for this is the poor quality of the surface signals. Surface EEnG studies [3,15,16,17,18,19], reported low amplitude of the intestinal signal picked up on the abdominal surface (above and below navel) and the presence of different types of interference such as low frequency drifts, respiratory interference (whose spectrum can sometimes overlap with that of the SW [16,18]) and cardiac interference (whose spectrum can overlap with that of the SB). In addition, all these studies focus on the SW, since the energy of the SB is more attenuated due to the effect of low-pass filtering associated with the abdominal layers and the distance between the signal source and the pickup electrodes.

It should be highlighted that, as in other bioelectrical recording applications, the signals are sensed by conventional disc electrodes in most cases. As mentioned in the previous section, the use of concentric ring electrodes can enhance the spatial resolution and signal quality. In fact, in surface EEnG studies these electrodes have proven to be more immune to cardiac and respiratory interference [3,18,19]. However, there is no agreed criteria on the size of the electrodes: ring electrodes have been tested with an external diameter of 24 mm and 37 mm [18,19]. Both these studies took dry recordings and reported high contact impedance and the influence of signal drifts, indicating the need for electrolytic gel in future studies. The electrodes should also be self-adhesive to facilitate their use and to provide a ‘track’ for gel deposition. Regarding the configuration of the concentric ring electrode, given the low amplitude of intestinal signals on the abdominal surface, bipolar or even monopolar records may be recommended (the latter not being a direct estimation of the Laplacian potential). The second goal of this work was to determine the best recording setup for intestinal SW identification in terms of: configuration (monopolar/bipolar records), ring electrode size, adhesive material and electrode location.

This paper is organized as follows: Section 2 describes the materials and methods, the concentric electrode developed, the signal recording protocol, the parameters calculated and the proposed methodology for comparing and selecting the best combination of recording factors. Section 3 gives the results, recorded signals and characteristic parameters. In Section 4 the results are discussed and the conclusions are given in Section 5.

## 2. Materials and Methods

### 2.1. Sensing Part

**Figure 1:**
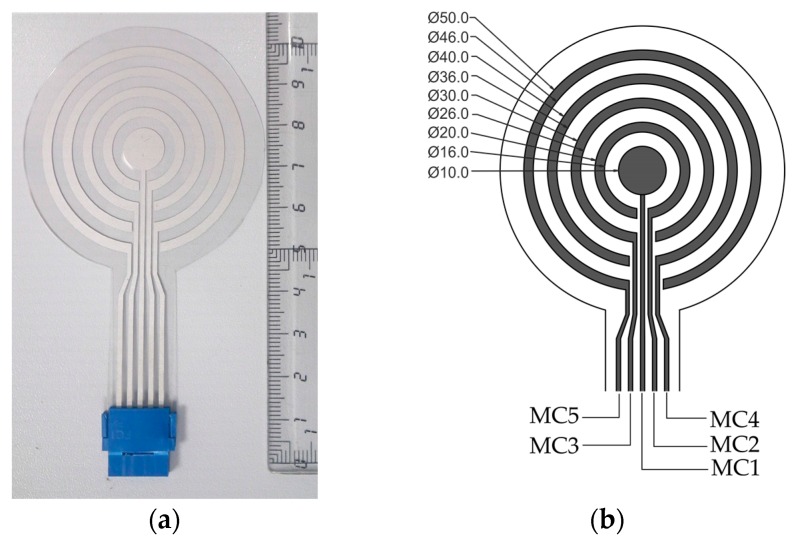
(**a**) Multi-ring concentric electrode; (**b**) Dimensions (in mm) of poles and annotation of signals sensed by each pole.

A multi-ring concentric ring electrode (multi-CRE) was designed as shown in Figure 1. The sensing electrode consists of a set of four hook-shaped electrodes and an inner circular electrode. This design allows direct connection of the inner disk and outer rings to the connector to the signal conditioning unit in a single conductive layer, and presents little deviance in spatial sensitivity to that of closed rings [20]. In this work, the diameter of the inner disc was set to 10 mm. The external ring diameter ranged from 20 mm to 50 mm, as shown in Figure 1b. The width of the rings was set to 2 mm. This provides sufficient contact area between the electrode and the skin and means the inter-ring distance can be set to 3 mm, which reduces the risk of undesired shortcuts between poles due to possible scattering of electrolytic gel in wet recordings.

In order to facilitate the adaptation of the electrode to a curved body surface and to make it more robust to interference and movement artifacts [21], the multi-CRE was implemented onto a flexible polyester substrate (Melinex ST506, 175 μm, DuPont, Chester, VA, USA).

Thick film serigraphic technology was used to produce the sensor. The screen printing process consists of forcing pastes of different characteristics through squeegees onto a substrate through screens. The gaps in the screen define the pattern that will be printed on the substrate by serigraphy. The final thickness of the pastes can be adjusted by varying the thickness of the screens. It is necessary to manufacture frames with screen mesh for each layer of the design. To build the electrode, one screen was made with 230 mesh polyester material (PET 1500 90/230-48 from Sefar, Heiden, Switzerland). A UV film Dirasol 132 (Fujifilm, Kansas, KS, USA) was used to transfer the stencil onto the screen mesh. Final screen thickness was 10 µm. The pattern was transferred onto the screen by a UV light source. The flexible electrodes were screen-printed with a biocompatible 80/20 Silver/Silver Chloride mix of Ag/Ag-Cl paste (C2130429D3, Gwent, Pontypool, UK). The main characteristics of this paste are shown in Table 1.

**Figure 2:**
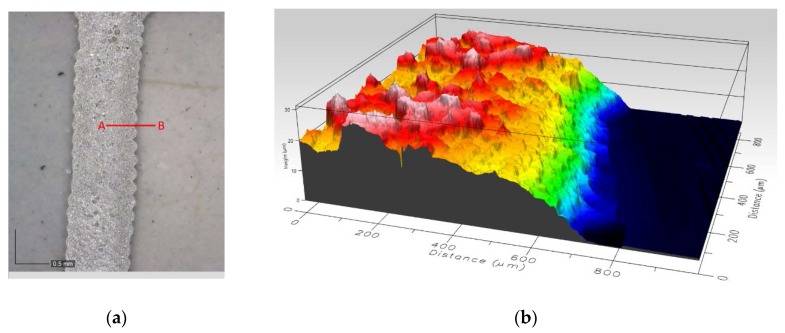
(**a**) Magnification view of a track of electrode; (**b**) Right: thickness of view A–B; average thickness is 20 µm measured by Profilm 3D (Filmetrics, San Diego, CA, USA) with 20× lens.

Printing was carried out by a high precision screen stencil printer (AUREL 900, Modigliana FC, Italy) with a 75° shore squeegee hardness, 3.5 bar force, and 8 mm/s. After paste depositing, this was cured in an air oven (UNB-100, Memmert GmbH+Co. KG, Schwabach, Germany) at 80 °C for 10 min. The ink curing period was 130 °C for 10 min. Figure 2 shows details of the results of the printing process.

This electrode design can yield signals with different configurations such as: monopolar concentric (MC), bipolar concentric (BC), tested in this work, and some others such as tripolar or quasi-bipolar configurations [2]. MC signals are the monopolar records obtained with the 5 poles of the electrode (see Figure 1b), with respect to a distant reference electrode. BC signals derived from multi-CRE are given by:

(1) $${\mathrm{BC}}_{n}{=\;\mathrm{MC}}_{n+1}-{\mathrm{MC}}_{1}$$

where,${\;\mathrm{MC}}_{n+1}$, n = 1…4 are the biopotentials picked up by the four rings from the inside out; ${\mathrm{MC}}_{1}$ is the biopotential picked up by the inner disc.

**Figure 3:**
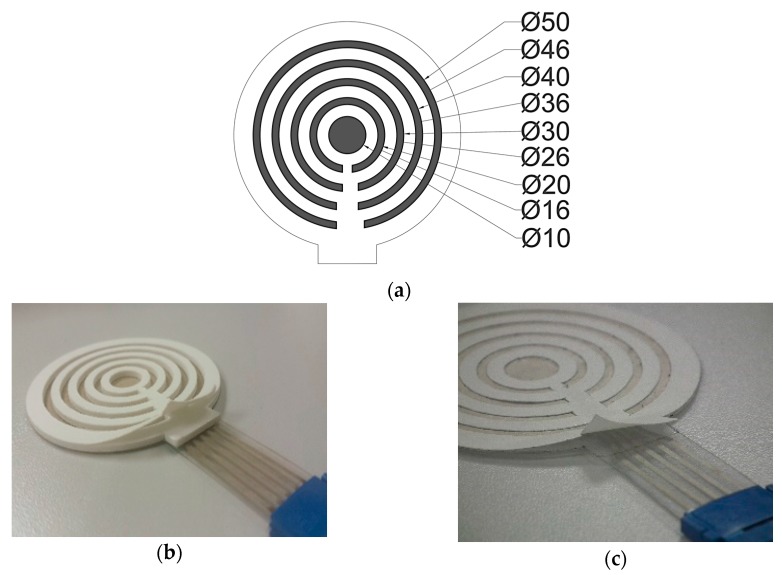
(**a**) Template design, size in mm; (**b**) Multi-CRE with foam; (**c**) Multi-CRE with adhesive.

In order to help the multi-CRE to stick to the skin and to create a gap for the deposition of the conductive gel between the electrode and skin, two types of solutions were used: adhesive only and adhesive+foam (hereinafter ‘adhesive’ and ‘foam’). The foam (ethylvinyl acetate) is a non-toxic polymer widely used in electrodes for the uptake of biosignals [22,23]. It can provide the appropriate stiffness while adapting to the curvature of the body and provides electrode stability to sudden movements (coughing, sneezing, involuntary movements of the subject) [23,24,25]. Its 1 mm thickness can deposit a thicker gel layer. The foam material does not have adherent properties; this is given by a second adhesive material, a double-sided adhesive (TM8710, MacTac, Soignies, Belgium) made of polyethylene terephthalate (PET) designed to adhere medical products to human skin. The template designed for the die-cutting of foam and adhesive materials and the implemented electrode with both adhesive materials are shown in Figure 3.

### 2.2. Recording Sessions

This study was approved by the Polytechnic University Ethics Committee and adhered to the Declaration of Helsinki. The subjects were informed of the nature of the study, briefed on the recording protocol and signed a consent form. Twenty recording sessions were performed on twenty healthy volunteers, ten for each type of foam and adhesive material. To study the effect of the recording position, EEnG records were made at both the above navel (AN) and below navel (BN) sites, with each volunteer in a supine position (30° from the horizontal). Firstly, basic biostatistical parameters such as weight, height, sex, surgical interventions relevant to the study and any history of gastrointestinal, cardiovascular or infectious disorders was noted. The skin was then prepared (exfoliation using abrasive pads and cleaning with 96% alcohol) in the areas under the electrodes at the level of the right and left middle clavicle (ECG recordings), AN and BN abdominal region (EEnG recordings) and the right ankle and left hip (reference and ground electrodes). The subjects were also shaved if hairs were present in these areas.

**Figure 4:**
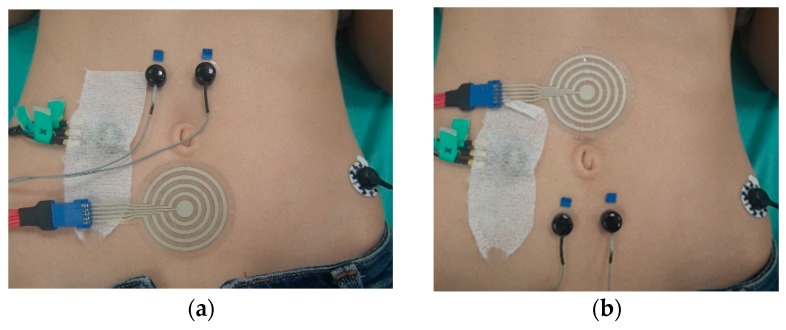
Location of electrodes and accelerometer (covered by adhesive tape). (**a**) Multi-CRE in BN position and conventional bipolar recording in AN position, reference electrode for monopolar measurements on left hip; (**b**) multi-CRE in AN position and conventional bipolar recording in BN position.

The conductive gel was spread on the multi-CRE without removing the adhesive backing of either material, spreading the gel carefully over all the rings. The adhesive backing was then removed and the electrode placed 2.5 cm above or below the navel (AN or BN positions, respectively), as shown in Figure 4a,b. Two monopolar Ag/Ag-Cl 8 mm recording diameter disk electrodes with 2.5 cm separations were placed 2.5 cm above or below the umbilicus to obtain one bipolar conventional EEnG recording. The duration of the recording sessions was 1 h with the multi-CRE in the BN (best expected location) and 10 min in the AN position.

The main sources of common types of physiological interference in surface EEnG recordings were also noted: ECG was recorded in the ML-Lead-I using disposable electrodes; respiration was measured with an airflow transducer (1401G Grass Technologies, Warwick, RI, USA) and movements were sensed by a triaxial accelerometer (ADXL 335, Analog Devices, Norwood, MA, USA). In order to help the multi-CRE to stick to the skin and to create a gap for the deposition of the conductive gel between the electrode and skin, two types of solutions were used: adhesive only and adhesive+foam (hereinafter ‘adhesive’ and ‘foam’). The foam (ethylvinyl acetate) is a non-toxic polymer widely used in electrodes for the uptake of biosignals [22,23]. It can provide the appropriate stiffness while adapting to the curvature of the body and provides electrode stability to sudden movements (coughing, sneezing, involuntary movements of the subject) [23,24,25]. Its 1 mm thickness can deposit a thicker gel layer. The foam material does not have adherent properties; this is given by a second adhesive material, a double-sided adhesive (TM8710, MacTac, Soignies, Belgium) made of polyethylene terephthalate (PET) designed to adhere medical products to human skin.

The template designed for the die-cutting of foam and adhesive materials and the implemented electrode with both adhesive materials are shown in Figure 3. A disposable electrode was placed on the right ankle and used as the bioelectric ground. Another electrode was placed on the left hip and used as reference potential for EEnG monopolar records.

All signals except those from the accelerometer were amplified and band-pass filtered (0.1–100 Hz) by means of conventional bioamplifiers (P511, Grass Technologies). Signals were simultaneously recorded at a sampling rate of 1 kHz.

### 2.3. Signal Analysis

In order to study the effect of the material, position, configuration and dimensions of the electrode rings on the detection of SW, ten EEnG signals were analyzed in each session: five monopolar concentric (MC-EEnG), four bipolar concentric (BC-EEnG) and one conventional bipolar (BIP). Artifacted signal segments were identified by accelerometer signals and visual inspection and were removed from the analysis (<3% of total windows). The EEnG signals and respiration signal were digitally low-pass filtered (fc = 0.5 Hz) and resampled at 4 Hz. The power spectral density (PSD) of these signals was estimated by means of autoregressive parametric techniques (AR, order 120) calculated in moving 120 s windows every 15 s. The dominant frequency (DF) was calculated in each moving window, being defined as the frequency of the maximum energy peak above 6 cpm. The signal quality in terms of respiration interference and low frequency components was also evaluated, calculating the Welch Periodogram for each moving window so as to compute subband energies. The following parameters were calculated from the obtained PSDs [19]:

- %DF_TFSW_: defined as the ratio of analyzed windows whose DF is inside the typical frequency range of intestinal SW (8–12 cpm).
- %DF_RESP_: defined as the ratio between the number of windows in which the DF of the surface signal is within the DF of respiration (DF_RESP_) ±1 cpm and the total number of windows
- PR_SW/RESP_: defined as the ratio between the power within the SW frequency range and the power in the respiratory bandwidth, calculated as follows:
  (2) $${\mathrm{PR}}_{\mathrm{SW}/\mathrm{RESP}}\left(\mathrm{dB}\right)\;=\;10\cdot \log\left(\frac{{\mathrm{Power}(\mathrm{EEnG})|}_{8\;\mathrm{cpm}}^{12\;\mathrm{cpm}}}{{\mathrm{Power}(\mathrm{EEnG})|}_{{\mathrm{DF}}_{\mathrm{RESP}}-1\;\mathrm{cpm}}^{{\mathrm{DF}}_{\mathrm{RESP}}+1\;\mathrm{cpm}}}\right)$$
- %DF_LF_: defined as the ratio between the number of windows in which the DF is within 6–8 cpm and the total number of windows.
- PR_SW/LF_: defined as the ratio between the power within the SW frequency range and the power in the low frequency bandwidth, calculated as follows:
  (3) $${\mathrm{PR}}_{\mathrm{SW}/\mathrm{LF}}\left(\mathrm{dB}\right)\;=\;10\cdot \log\left(\frac{{\mathrm{Power}(\mathrm{EEnG})|}_{8\;\mathrm{cpm}}^{12\;\mathrm{cpm}}}{{\mathrm{Power}(\mathrm{EEnG})|}_{6\;\mathrm{cpm}}^{8\;\mathrm{cpm}}}\right)$$
- %DF_OTHERS_: defined as the ratio between the number of other cases and the total number of windows. Ideally, this parameter should be 0%.
- %DF_SW_: defined as the ratio of analyzed windows whose DF after discarding peaks on the low frequency and respiration bandwidth is in the SW range.
- PR_ECG_: defined as the ratio between the difference between the power of the raw signal (${\mathrm{Power}}_{\mathrm{EEnG}}$) and the power of the ECG interference estimation (${\mathrm{Power}}_{I_{\mathrm{ECG}}}$). The power of the ECG interference estimation was worked out as described in [26,27]:
  (4) $${\mathrm{PR}}_{\mathrm{ECG}}\left(\mathrm{dB}\right)\;=\;10\cdot \log\left(\frac{{\mathrm{Power}}_{\mathrm{EEnG}}\;{-\;\mathrm{Power}}_{I_{\mathrm{ECG}}}}{{\mathrm{Power}}_{I_{\mathrm{ECG}}}}\right)$$
- %RS (rhythm stability): which is the percentage of the recording session windows in which DF are within the range of mean(DF) ± 2cpm. This interval is considered an acceptable frequency change for the SW component of the EEnG in humans [3,28].
- MV (mean variability): defined as the average of the dominant frequency difference between consecutive windows in the range of 8 to 12 cpm, where ‘R_i_’ is the dominant frequency of the EEnG signal in the window ‘i’ and ‘N’ is the number of analyzed windows in the session:
  (5) $$\mathrm{MV}\;=\;\frac{1}{N}\overset{N}{\underset{i=1}{\sum }}\left|R_{i+1}\;{-\;R}_{i}\right|$$

### 2.4. Selecting the Best Combination of Recording Factors of CRE

Eight combinations of three signal recording factors were analyzed: configuration (MC, BC), material (adhesive, foam) and position (AN, BN). It should be noted that for the MC and BC configurations, the parameters obtained for the five MC and four BC channels were averaged, respectively. The influence of the electrode size was studied in a subsequent approach.

In order to determine the best combination of factors for recording the EEnG signal on the body surface, an Improvement Ratio of a given parameter ‘Y’ (IR_Y_) was proposed and calculated, defined in such a way that its value varies between ‘1’ for the combination of factors that yields the ‘best’ average (N = 10 subjects) value of ‘Y’ parameter, and ‘0’ for the worst of the 8 factor combinations. The ‘best’ result for a parameter is its maximum value if it is a beneficial parameter i.e., a higher signal/interference ratio, and is its minimum value if it is a detrimental to signal quality, i.e., a higher percentage of dominant frequencies associated with an interference. According to this, the improvement ratio of a given parameter is calculated as follows:

(6) $${\mathrm{IR}}_{\mathrm{Yi}}\;=\frac{M_{\mathrm{Yi}}\;-\;\min\left\{M_{\mathrm{Yi}}\right\}}{\max\left\{M_{\mathrm{Yi}}\right\}\;-\;\min\left\{M_{\mathrm{Yi}}\right\}}$$

where Y = 1…6 are beneficial parameters (see Table 2); i = 1 … 8, ‘i’ being each combination of factors; M_Yi_ is the mean of parameter ‘Y’ for combination ‘i’;

(7) $${\mathrm{IR}}_{\mathrm{Yi}}\;=\frac{\max\left\{M_{\mathrm{Yi}}\right\}-M_{\mathrm{Yi}}}{\max\left\{M_{\mathrm{Yi}}\right\}-\min\left\{M_{\mathrm{Yi}}\right\}}$$

where Y = 7…10 are detrimental parameters (see Table 2); i = 1 … 8, ‘i’ being each combination of factors; M_Yi_ is the mean of parameter ‘Y’ for combination ‘i’.

In order to determine whether a combination of factors globally improves or worsens EEnG signal quality and the capacity to detect intestinal SW, a weight (W) was assigned to each improvement ratio parameter (${\mathrm{IR}}_{\mathrm{Yi}})$. The value of the weight was assigned according to the research group’s experience of the greater or lesser relevance of the parameter on EEnG recordings. The weights associated with each parameter are shown in Table 2. The highest weight (0.25) is assigned to %DF_TFSW_, since it is the most frequently used parameter in the literature for the identification of intestinal SW activity [16]. The main types of interference, such as respiration and low frequency, were equally weighted (0.125), 0.0625 then being the weight of each of the two parameters that value each type of interference (%DF_RESP_, PR_SW/RESP_, %DF_LF_, PR_SW/LF_). The same weight (0.125) was assigned to parameters that assess the rhythmicity of the dominant frequency, 0.0625 for %RS and for MV. The parameter %DF_SW_ was assigned with half of the %DF_TFSW_ weight (0.125) because its value is conditioned by discarding interferences; %DF_OTHERS_ is also weighted by the same value (0.125) since the combinations with the smallest values of this parameter are of interest. Finally, PR_ECG_ was weighted by 0.125. Although the ECG is outside the range of the SW and the weights are focused on SW detection, using the electrode to detect intestinal bursts is not ruled out. In this case it may also be important that the electrode attenuate cardiac interference.

Finally, for each combination of factors ‘i’, a global improvement index ${\mathrm{IRglobal}}_{i}$ is computed as a weighted sum of the IR of the 10 parameters studied:

(8) $${\mathrm{IRglobal}}_{i}=\overset{10}{\underset{Y=1}{\sum }}{(W}_{Y}{}^{*}{\mathrm{IR}}_{\mathrm{Yi}})$$

IR_global_ will assess how close combination ‘i’ would be to an ideal case using the optimal combination of each of the parameters tested. ‘1’ would be optimal and ‘0’ the worst, i.e., always use the worst possible combination for each parameter.

### 2.5. Selecting the Best CRE Ring Size and Comparison with Conventional Bipolar Recording

After determining the most suitable combination of factors for picking up the surface EEnG signal by CRE had been determined, the effect of ring size was studied. The parameters associated with the bipolar recording obtained with conventional disc electrodes (BIP) were also part of the group studied to look for the most appropriate way to record EEnG signals. Signal quality and capacity to identify SW frequency was evaluated and compared by means of improvement ratios, as described above.

## 3. Results

### 3.1. Signal Acquisition and Parameters

**Figure 5:**
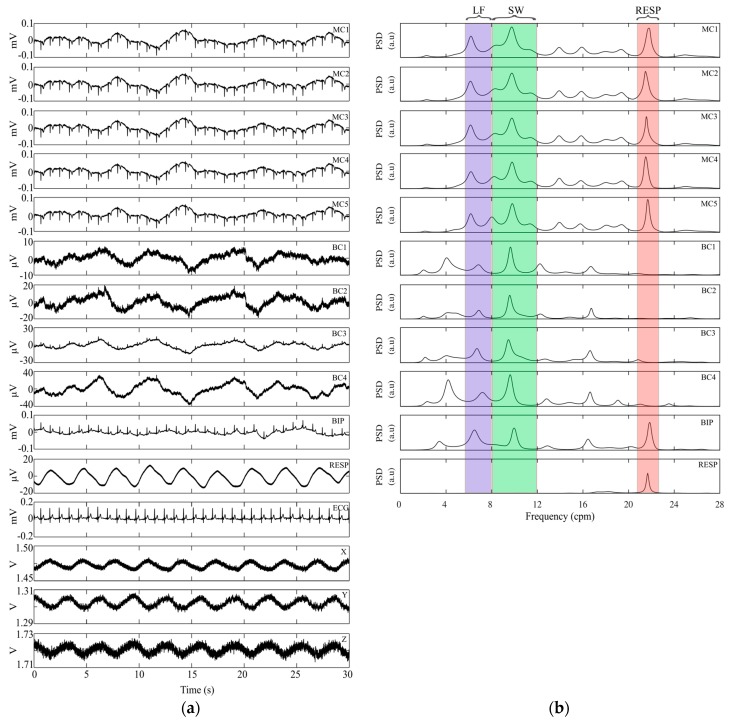
(**a**) Thirty seconds of signals: monopolar concentric (MC1-5), bipolar concentric (BC1-4) with adhesive material below navel position; bipolar (BIP), respiration (RESP), ECG ML-Lead I and accelerometer signals (X, Y, Z); (**b**) Power spectral density of signals shown on the left.

Figure 5 shows an example of the signals recorded and their PSDs. It can be seen that the amplitude of the MC-EEnG signals does not change significantly for different ring sizes. However, the larger the size of the ring, the larger the amplitude of the BC-EEnG signal. It can also be seen that ECG interference was higher in MC-EEnG and BIP than BC-EEnG signals, as corroborated by the results of the PR_ECG_ parameter shown in Table 3 and Table 4.

Visual identification of the slow wave in the time domain is not clear, mainly due to cardiac and respiratory interference in MC-EEnG and BIP records, and to the low amplitude in BC-EEnG records. Figure 5b shows the PSD of the corresponding temporal signals in analysis windows of 120 s after low-pass filtering and resampling. It can be seen that in BC-EEnG records the highest energy peaks and the associated DFs are clearly in the SW frequency range (8–12 cpm), while the respiratory interference is more manifest in MC-EEnG and BIP and maximum energy peaks could be obtained in the respiratory frequency. The better immunity of bipolar concentric configuration to respiratory interference is confirmed by the results from parameters %DF_RESP_ and PR_SW/RESP_, as shown in Table 3 and Table 4. Higher signal-to-respiratory interference ratios were obtained for BC-EEnG than MC-EEnG and BIP; the DF frequencies were also associated with respiratory interference in a smaller number of cases. However, the opposite behavior is seen in the influence of low frequency interference. The signal-to-low frequency interference ratio was lower and %DF_LF_ higher, for BC-EEnG than for MC-EEnG or BIP, as shown in Table 3 and Table 4. Overall, the best %DF_SW_ results were obtained with the BC configuration, at mean values of 93% in the BN position, which drops to 85–87% in the AN position. In the RS and MV rhythmicity parameters, no clear superiority of any configuration or material was observed, with the exception of MC in BN and adhesive material, which proved to be the best combination in this context.

### 3.2. Selection of the Best Combination of CRE Recording Factors

Table 5 shows the improvement ratio of each combination of factors for the different parameters. The BN provides clearly better results than AN for the ratio of cases in which ‘raw’ signal DF could be attributed to the SW frequency (%DF_TFSW_) when foam was used and very similar ones for adhesive. BN is also clearly better after discarding the energy peaks associated with possible interference (%DF_TFSW_). The highest immunity to respiratory interference is obtained by BN plus adhesive. The best behavior in terms of robustness to low frequency and cardiac interference is obtained by the MC and BC configurations, respectively.

**Figure 6:**
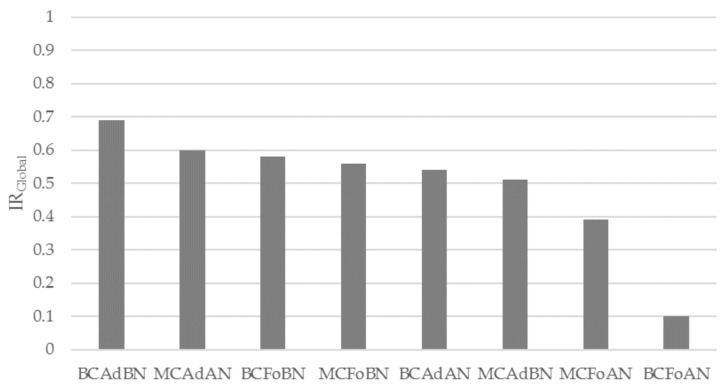
IR_Global_ for each combination of factors: configuration (MC: monopolar concentric; BC: bipolar concentric), material (Ad: adhesive; Fo: foam) and position (AN: above navel; BN: below navel).

The color scheme in Table 5 shows the best and worst combinations when considering all parameters in a global approach. This is clearest in the IR_Global_ values given in Figure 6. It can be seen that in general, the lowest IRs and IR_Global_ are from the records from AN with foam for both MC and BC (BCFoAN and MCFoAN). The best IRs seem to concentrate in the combination of BC with adhesive and BN ition(BCAdBN), which obtained the highest IR_Global_ values (0.7), followed by MCAdBN and BCFoBN, with values around 0.6.

### 3.3. Selection of the Best Ring Size of CRE and Comparison with Conventional Bipolar Recording

After identifying the best combination of CRE recording factors to capture the SW, the next step was to study the influence of ring dimensions on the values of the characteristic EEnG parameters. The influence of the electrode dimensions was analyzed for the BC-EEnG configuration in BN position plus adhesive. The characteristic parameters of the four bipolar concentric signals (BCn-EEnG, *n* = 1.4) of Ø20, Ø30, Ø40, Ø50 mm outer ring diameters, respectively, were studied and the best choice was determined in the same way as in the previous step. The results obtained were compared with those of conventional bipolar registers by means of cup disc electrodes at the same BN position.

**Figure 7:**
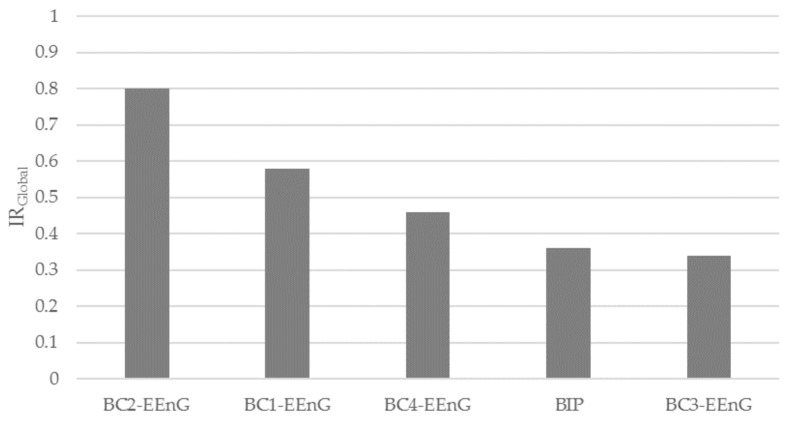
IR_Global_ for each concentric bipolar configuration (BC1-EEnG, BC2-EEnG, BC3-EEnG and BC4-EEnG) and conventional bipolar (BIP).

Table 6 shows the IR values of each BC-EEnG signal and BIP. It can be seen that the IRs of the BIP were lowest for most parameters, except for those associated with immunity to low-frequency interference. It is also noticeable that for BC-EEnG signals, the larger the electrode the worse the immunity to respiratory interference, and the better to low frequency interference. It seems that the best compromise is found for BC2-EEnG (Ø30 mm), whose IR values tend to be highest. These results agree with those given in Figure 7, which represents the IR_Global_ values. According to the selection criteria, the optimal diameter of the concentric electrode ring for SW identification in surface EEnG signals is 30 mm; which belonged to BC2-EEnG with an IR_Global_ of 0.80.

## 4. Discussion

The multi-CRE developed in this work was implemented onto a flexible substrate and had an adhesive layer to fix it to the abdomen. It was comfortable for the patient, adapted to fit the body surface and could be easily handled by the technician. The fact that it had only a single connector reduced the possibility of incorrect connections and simplified the protocol.

The electrode was produced by screen printing technology. At present, several different graphic art printing techniques are available to make electronic devices, sensors or electrodes. The most widely used methods are screen printing, inkjet printing, stamping/nanoimprinting, and gravure printing. Previous studies compared some of these techniques for the implementation of flexible CRE [29]. The results obtained for ink thickness, resistivity, electrode resistance, and other performance parameters derived from electrocardiographic signal recording tests suggest that screen-printing and inkjets are suitable for non-invasive bioelectric signal acquisition. In the present work, the inkjet printing option was discarded since it is less robust in terms of conductive ink fixation, i.e., the ink tends to peel off when removing the adhesive.

The use of the developed multi-CRE for non-invasive EEnG recording permitted the study of the influence of the factors affecting recording conditions on different signal quality parameters of this specific application. In a preliminary work, we computed a reduced set of parameters (%DF_TFSW,_ PR_RESP_, PR_LF_ and %DF_SW_) for different ring sizes and configurations with multi-CRE in below navel position and adhesive material [30]. In the present work, factors such as the recording position and the type of adhesive material have also been considered, and its effects has been studied for a more comprehensive set of 10 parameters. It should be noted that we did not specifically study the influence of recording factors on the signal amplitude, since it is strongly influenced by interference such as respiration and the ECG. As these types of interference can be greater than the intestinal signal itself [16,18,31], the amplitudes captured on the surface cannot be attributed solely to intestinal myoelectric activity, which would need to be eliminated in order to determine the EEnG amplitude. It was observed that amplitude usually increases with ring size for monopolar and bipolar concentric records, especially with adhesive and regardless of the position. This agrees with previous studies on other bioelectric signals that report larger amplitudes for larger rings, although without significant differences [32,33,34,35] and in finite element models [8].

The best results were obtained from the BN position as AN was more affected by respiratory and cardiac interference. In this position the electrode is closer to the thoracic cage and the diaphragm, and thus more liable to respiratory movement. It is also closer to the heart, and therefore cardiac interference is also stronger. In the BN there were fewer cases where the DF did not correspond to the range defined for SW or its main types of interference. All this leads to better SW identification in the BN, especially after discarding undesired energy peaks, in agreement with previous studies [18], given that there is a greater concentration of intestinal loops in this area and it is further away from the stomach and diaphragm, which can generate high gastric and respiratory interference.

Of the materials used to fix the electrode in place, i.e., adhesive only or adhesive with foam, the former showed better behavior in terms of robustness to respiratory and low frequency interference. As seen in other studies, respiratory interference in surface records is due to a great extent to the potential difference between the electrodes due to physical movements [16,18,31], so that the greater rigidity introduced by the foam could be responsible for this higher respiratory interference. It could also lead to poor skin contact, increasing electrode-skin impedance [36,37] and in turn increasing low frequency interference. Also, foam leaves a larger volume of gel on the multi-CRE, which may not be evenly distributed and thus could lead to poorer results. However, this could be solved by using solid electrolyte gel or hydrogel, which would achieve two important improvements: better skin contact and the fixation would be self-adhesive, since many solid conductor gels are also adhesives, although it would also increase costs.

With respect to the configuration of the signal recording, BC proved to be better than MC in terms of attenuation of respiratory and cardiac interference. Both types of interference are coupled to the signal as common mode interference, i.e., with similar morphology and amplitude in the different poles of the multi-CRE. Therefore, they are significantly reduced in BCs, which are obtained by subtracting the potential sensed by the central disk with that of the outer rings. In fact, most studies with concentric ring electrodes perform bipolar records [18,19,38], while it has been found that low frequency interference affects the EEnG signals in the BC configuration more than MC regardless of the electrode position and material. This seems to indicate that the low frequency interference, like fluctuations of the contact potential or baseline are not similar (common mode component) between both poles of the record or do not cancel, in fact, they increase slightly, when computing the potential difference between both. The different behavior of the BC signals could also be due to the fact that the amplitude of the signals are smaller, increasing the effect of this interference.

It should be mentioned that some authors have also proposed other recording configurations with multipole concentric ring electrodes for a more accurate estimation of the Laplacian potential, such as a tripolar configuration (experimentally tested) [39] and even a generalized approach for n rings (at a theoretical level) [40]. These other configurations lie beyond the scope of the present work, but they could be worked out with the electrode developed in the present study.

All in all, the proposed methodology based on the definition of improvement ratios found that when considering all parameters in a global approach the best combination of factors was a bipolar concentric configuration with adhesive material in the BN position. With this combination of factors, the influence of the ring size was studied and the results were compared with conventional bipolar recordings with disk electrodes. Theoretically, smaller rings capture dipole sources closer to the body surface and the larger the ring the deeper the activity that can be sensed. However, the larger the diameter, the greater the averaging effect of different dipole sources and the lower the spatial resolution.

It was observed that larger ring sizes have less low frequency interference. Since all the rings were 2 mm wide, this behavior could be due to the larger skin-to-electrode contact area and the associated reduced contact impedance. On the other hand, the influence of respiratory interference on the EEnG record is greater with increasing ring size, probably because the possible movements of the central disk and the outer ring caused by respiration are less temporarily synchronized by the larger outer ring [30]. The best results in terms of intestinal SW identification and global signal quality were obtained with the 30 mm outer diameter ring, which manages to balance the previously indicated factors. Some authors have proposed the used of ring electrodes to pick up other bioelectric surface signals with similar dimensions to those analyzed in the present work. For instance Ye-Lin et al. pointed out the feasibility of capturing uterine myoelectric activity in at-term pregnant women by 24 mm diameter concentric bipolar electrodes [32]. Also in the field of electrocardiography, Prats et al. and Lu et al. observed that with concentric rings of 33.5 mm and 36 mm a compromise was reached between the better usability and spatial resolution of small rings and the larger signal amplitudes achieved by larger electrodes [5,41].

Lastly, comparing the signals picked up by the multi-CRE with those from conventional bipolar recordings by disc electrodes, bipolar concentric records presented greater attenuation of cardiac interference. These results agree with other studies in which bipolar configuration concentric ring electrodes were used to sense surface myoelectric activity from different organs and muscles, such as the small bowel (in quasi-bipolar configuration) [18,19], the uterus [38], the diaphragm [42] and the sternocleidomastoid [43]. This behavior could be mainly attributed to the enhanced spatial resolution of bipolar concentric electrodes, which are more sensitive to the activity of dipole sources close to the electrode and less sensitive to distant dipoles such as those of cardiac origin. On the other hand, unlike conventional bipolar records, BC electrodes attenuate respiratory interference. This is probably due to the more homogeneous movement of the central disc and the outer ring combined on a single electrode, than that of the two independent disc electrodes used for conventional bipolar records. However, the latter outperform the BC in terms of the influence of low frequency interference. All in all, in identifying the SW frequency and obtaining surface EEnG signals, the multi-CRE developed for this study in general achieved better results.

## 5. Conclusions

In this work a multipole concentric ring electrode was developed to perform simultaneous recordings with different ring sizes and configurations (monopolar, bipolar, tripolar, etc.). To facilitate its clinical use and improve its body contact and comfort, it was implemented onto a flexible substrate and incorporated an adhesive layer and a single output connector.

This electrode can provide the best signal-sensing arrangement for each surface bioelectrical recording application. The factors evaluated were: the effect of the electrode position, adhesive material, configuration and ring size on signal quality and intestinal SW detection capacity parameters derived from surface EEnG recordings.

The proposed methodology for the evaluation of the different signal sensing arrangements based on the definition of improvement ratios found that for this specific application the best combination of factors was: bipolar concentric recordings below the navel with an adhesive material without foam, and a ring size of 30mm outer diameter. The signals derived from the multi-CRE yielded signals of better quality and were better able to identify intestinal SW than conventional bipolar records with disc electrodes.

The developed multi-CRE and proposed methodology for the comparison of alternatives could thus be used to find the most appropriate signal sensing arrangement for other biomedical applications providing non-invasive bioelectrical recordings from other organs and muscles, such as the heart, brain, uterus, respiratory muscles, etc.

## Figures and Tables

**Table 1:** C2130429D3 ink’s parameters.

Property	Value
Solids Contents (%)	81.14–83.14
Vicosity (Pa.s)	2.0–5.5
Curing condition (°C)	80 °C/10 min
Sheet resistivity (25 µm)	< 30 mΩ/sq

**Table 2:** Weights for the improvement ratios of parameters involved in the selection of the most appropriate combination of factors.

Y	Improvement Ratio (IR_Y_)	Weight (W_Y_)
1	%DF_TFSW_	0.25
2	%DF_SW_	0.125
3	PR_ECG_	0.125
4	PR_SW/RESP_	0.0625
5	PR_SW/LF_	0.0625
6	%RS	0.0625
7	%DF_RESP_	0.0625
8	%DF_LF_	0.0625
9	%DF_OTHERS_	0.125
10	MV	0.0625

**Table 3:** Mean and standard deviation of the parameters defined for SW identification and interference quantification of surface abdominal signals picked up with different recording factors. Signals recorded in BN position in 10 volunteers.

	MC-EEnG (*n* = 50)		BC-EEnG (*n* = 40)		BIP (*n* = 10)	
	Foam	Adh	Foam	Adh	Foam	Adh
**%DF_TFSW_ (%)**	56.0 ± 12.6	56.1 ± 9.5	54.1 ± 8.3	57.3 ± 12.7	53.4 ± 17.2	58.7 ± 11.5
**%DF_RESP_ (%)**	19.7 ± 14.1	16.1 ± 9.2	14.6 ± 9.8	14.4 ± 11.2	23.9 ± 22.1	21.7 ± 9.5
**PR_SW/RESP_ (dB)**	5.01 ± 1.82	4.74 ± 1.74	5.59 ± 1.76	5.19 ± 2.20	3.81 ± 2.03	4.07 ± 2.21
**%DF_LF_ (%)**	16.6 ± 7.1	16.7 ± 7.6	24.6 ± 10.0	21.6 ± 9.3	12.5 ± 11.3	9.6 ± 3.8
**PR_SW/LF_ (dB)**	3.78 ± 1.44	3.86 ± 0.92	2.86 ± 1.45	3.55 ± 1.25	4.25 ± 1.58	4.97 ± 1.47
**%DF_OTHERS_ (%)**	7.9 ± 4.3	11.1 ± 5.5	6.7 ± 5.3	6.7 ± 3.9	10.2 ± 12.9	9.9 ± 8.2
**PR_ECG_ (dB)**	6.39 ± 3.89	6.13 ± 1.82	11.17 ± 4.32	19.77 ± 4.90	7.93 ± 4.91	6.57 ± 3.55
**%DF_SW_ (%)**	92.1 ± 4.2	88.8 ± 5.5	93.2 ± 5.3	93.2 ± 3.9	87.0 ± 12.1	89.6 ± 8.5
**DF_SW_ (cpm)**	9.97 ± 0.26	9.84 ± 0.35	9.99 ± 0.21	9.72 ± 0.49	9.49 ± 0.48	9.64 ± 0.20
**RS (%)**	58.9 ± 15.5	54.5 ± 21.7	58.8 ± 14.3	56.4 ± 22.8	60.9 ± 26.3	72.6 ± 28.8
**MV (cpm)**	0.38 ± 0.07	0.39 ± 0.10	0.41 ± 0.09	0.40 ± 0.11	0.38 ± 0.23	0.30 ± 0.26

**Table 4:** Mean and standard deviation of the parameters defined for SW identification and interference quantification of surface abdominal signals picked up with different recording factors. Signals recorded in AN position in 10 volunteers.

	MC-EEnG (*n* = 50)		BC-EEnG (*n* = 40)		BIP (*n* = 10)	
	Foam	Adh	Foam	Adh	Foam	Adh
**%DF_TFSW_ (%)**	45.9 ± 21.0	57.8 ± 14.9	32.9 ± 11.4	57.0 ± 12.5	49.3 ± 10.7	50.1 ± 10.2
**%DF_RESP_ (%)**	25.9 ± 23.0	19.6 ± 18.1	24.8 ± 18.4	12.3 ± 11.1	29.8 ± 14.3	25.6 ± 14.0
**PR_SW/RESP_ (dB)**	4.23 ± 2.92	4.14 ± 2.13	2.58 ± 1.77	4.98 ± 2.18	3.83 ± 1.83	3.51 ± 1.94
**%DF_LF_ (%)**	18.8 ± 12.4	8.8 ± 5.9	29.8 ± 16.3	15.9 ± 10.1	13.5 ± 8.1	14.1 ± 8.6
**PR_SW/LF_ (dB)**	3.69 ± 1.90	5.15 ± 0.81	2.11 ± 1.55	4.16 ± 1.84	5.16 ± 1.26	4.63 ± 1.37
**%DF_OTHERS_(%)**	9.40 ± 8.6	13.8 ± 7.8	12.4 ± 8.4	14.8 ± 10.2	7.4 ± 4.4	10.2 ± 8.8
**PR_ECG_(dB)**	7.82 ± 3.96	4.44 ± 2.94	11.83 ± 6.65	14.43 ± 6.17	6.13 ± 4.49	4.52 ± 2.03
**%DF_SW_(%)**	90.5 ± 8.6	86.1 ± 7.8	87.5 ± 8.4	85.1 ± 10.2	89.4 ± 4.3	88.5 ± 8.8
**DF_SW_(cpm)**	9.98 ± 0.32	10.29 ± 0.70	9.69 ± 0.51	9.69 ± 0.55	10.04 ± 0.26	9.59 ± 0.28
**RS (%)**	52.8 ± 29.2	80.1 ± 20.1	40.3 ± 30.0	54.5 ± 30.4	56.1 ± 10.7	47.5 ± 23.6
**MV (cpm)**	0.35 ± 0.16	0.34 ± 0.21	0.46 ± 0.11	0.44 ± 0.15	0.49 ± 0.11	0.37 ± 0.08

**Table 5:** IR values for the eight combinations of recordings factors and for the 10 parameters defined.

	Below Navel				Above Navel			
	Foam		Adhesive		Foam		Adhesive	
	BC	MC	BC	MC	BC	MC	BC	MC
	BCFoBN	MCFoBN	BCAdBN	MCAdBN	BCFoAN	MCFoAN	BCAdAN	MCAdAN
**%DF_TFSW_**	0.84	0.92	0.97	0.93	0.00	0.51	0.96	1.00
**%DF_RESP_**	0.83	0.46	0.84	0.72	0.08	0.00	1.00	0.86
**PR_SW/RESP_**	1.00	0.81	0.86	0.72	0.00	0.54	0.79	0.52
**%DF_LF_**	0.24	0.62	0.39	0.62	0.00	0.52	0.66	1.00
**PR_SW/LF_**	0.24	0.54	0.47	0.57	0.00	0.52	0.67	1.00
**%DF_OTHERS_**	1.00	0.85	0.99	0.45	0.29	0.66	0.00	0.11
**PR_ECG_**	0.44	0.12	1.00	0.11	0.48	0.22	0.65	0.00
**%DF_SW_**	1.00	0.87	0.99	0.45	0.29	0.66	0.00	0.11
**RS**	0.55	0.59	0.41	0.36	0.00	0.31	0.35	1.00
**MV (cpm)**	0.24	0.36	0.27	0.34	0.00	0.51	0.11	1.00
**IR_Global_**	0.58	0.56	0.69	0.51	0.10	0.39	0.54	0.60
							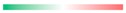	
							Best (1) Poor (0)	

**Table 6:** IR values for the concentric bipolar records (BC1-EEnG, BC2-EEnG, BC3-EEnG, BC4-EEnG) in BN position and using adhesive material and conventional bipolar (BIP) in BN position, for the 10 characteristic signal parameters.

	BC1-EEnG	BC2-EEnG	BC3-EEnG	BC4-EEnG	BIP
**%DF_TFSW_**	0.84	1.00	0.00	0.21	0.68
**%DF_RESP_**	1.00	0.83	0.35	0.00	0.05
**PR_RESP_**	1.00	0.91	0.19	0.05	0.00
**%DF_LF_**	0.00	0.09	0.19	0.59	1.00
**PR_LF_**	0.00	0.31	0.42	0.53	1.00
**%DF_OTHERS_**	0.51	1.00	0.45	0.59	0.00
**PR_ECG_**	0.67	0.91	0.47	1.00	0.00
**%DF_SW_**	0.53	1.00	0.49	0.62	0.00
**%RS**	0.14	0.38	1.00	0.92	0.00
**MV**	0.40	0.52	0.51	0.00	1.00
**IR_Global_**	0.58	0.80	0.34	0.46	0.36
				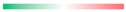	
				Best (1) Poor (0)	
